# Prelithiation of Alloy Anodes via Roll Pressing for Solid‐State Batteries

**DOI:** 10.1002/adma.202508973

**Published:** 2025-08-23

**Authors:** Congcheng Wang, Won Joon Jeong, Douglas Lars Nelson, Hari Sridhara, Sophia Nicolette Anderson, Matthew T. McDowell

**Affiliations:** ^1^ George W. Woodruff School of Mechanical Engineering Georgia Institute of Technology Atlanta GA 30332 USA; ^2^ School of Materials Science and Engineering Georgia Institute of Technology Atlanta GA 30332 USA

**Keywords:** alloy anode, electrochemistry, energy storage, prelithiation, solid‐state batteries

## Abstract

Solid‐state batteries with alloy‐type negative electrodes can feature enhanced energy density and safety compared to conventional Li‐ion batteries. However, diffusional Li trapping within Li alloys often causes low initial Coulombic efficiency and leads to capacity loss with cycling. Here, a general roll‐pressing prelithiation method compatible with a variety of alloy‐type negative electrodes (silicon, aluminum, tin, and multi‐phase alloys) is introduced, which is shown to improve performance in solid‐state batteries. By warm‐rolling lithium foil of controlled thickness with various alloy‐type electrodes, both slurry‐cast and foil‐type electrodes can be uniformly prelithiated via direct chemical reaction. The prelithiated electrodes exhibit enhanced specific capacity and extended cycle life in batteries with Li_6_PS_5_Cl solid‐state electrolyte. Prelithiated multi‐phase foil electrodes with a tailored interface are shown to exhibit superior cycling stability down to 2 MPa stack pressure. This prelithiation technique offers a pathway to overcome intrinsic challenges in alloy anodes for solid‐state batteries.

## Graphical abstract

A general prelithiation method is developed for alloy‐type negative electrodes via solid‐state lithiation using roll‐pressing with thin lithium foil. This mechanical prelithiation method is compatible with roll‐to‐roll processing and allows for a controlled extent of prelithiation. Prelithiated Si, Al, and Sn anodes, as well as multi‐component foil anodes, exhibit improved Coulombic efficiency and cycling stability in solid‐state batteries with sulfide‐based electrolytes.

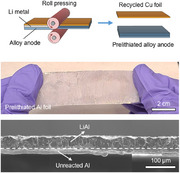

## 1 Introduction

The solid‐state battery (SSB) is an emerging technology that could be safer than conventional Li‐ion batteries while also allowing for the use of high‐capacity electrode materials to increase energy density.^[^ ^1^, ^2^, ^3^ ^]^ Research on SSBs largely focuses on either implementing pure Li metal anodes or composite anodes that contain alloy or other types of active materials mixed with inactive solid‐state ion‐conducting materials.^[^ ^4^, ^5^ ^]^ Li metal is a promising anode material, but it poses substantial challenges due to interfacial contact loss and short circuiting, which cause battery failure.^[^ ^4^, ^5^, ^6^ ^]^ Composite anodes that contain active materials mixed with solid‐state electrolyte (SSE) typically feature relatively low energy density because of the excess inactive materials present.^[^ ^7^, ^8^ ^]^

Recently, dense Al‐based foils^[^ ^9^, ^10^ ^]^ and SSE‐free particulate Si electrodes^[^ ^11^, ^12^, ^13^ ^]^ have drawn increasing attention as alloy anodes for SSBs.^[^ ^14^ ^]^ These materials exhibit promising performance while offering high energy density and low manufacturing cost. Furthermore, these dense alloy anodes feature a 2D interface with the SSE, which results in minimal formation of solid‐electrolyte interphase (SEI) compared to liquid‐electrolyte‐filled electrodes in Li‐ion batteries.^[^ ^2^, ^15^ ^]^ However, the lack of an SSE phase inside the electrode means that the active material itself must effectively transport Li during charge and discharge, and diffusional Li trapping in the bulk of these dense electrodes could cause low initial Coulombic efficiency (ICE) and capacity loss.^[^ ^10^ ^]^

Prelithiation is an effective way to address the issue of active Li loss and to increase the ICE of Li batteries.^[^ ^16^, ^17^ ^]^ Two typical strategies are electrochemical and chemical prelithiation.^[^ ^18^ ^]^ Electrochemical approaches involve the electrochemical lithiation reaction of the active material in temporary cells, which is usually time‐consuming and requires additional cell assembly and disassembly.^[^ ^16^ ^]^ Chemical prelithiation can be performed by immersing anode active materials in Li‐containing solutions,^[^ ^18^ ^]^ amongst other methods. The large‐scale usage of reactive Li‐containing solutions raises safety concerns, and such solutions may not react fully with dense foil‐type anodes. Moreover, both these prelithiation methods require complex cleaning and/or disassembly processes. As a result, they are limited by processing complexity and/or lack of universality, and they are difficult to incorporate into the widely used roll‐to‐roll manufacturing process.^[^ ^19^ ^]^

A few attempts have been made to develop mechanical prelithiation methods (i.e., direct chemical reaction with Li metal) for alloy anodes in SSBs^[^ ^20^, ^21^ ^]^ and Li‐ion batteries.^[^ ^22^ ^]^ Stabilized Li metal powder has been used as an additive agent for the prelithiation of Si particulate electrodes.^[^ ^20^, ^23^, ^24^ ^]^ High stack pressures are then applied to cause a reaction with the Li powder. However, stabilized Li metal powder usually has a relatively large particle size compared to the active materials, which could cause uneven lithiation and nonuniform distribution of the Li alloy phases, resulting in degraded performance. This strategy also suffers from the high cost and potential safety hazards of the Li powder. For foil‐type alloy anodes, the addition of a piece of Li metal foil onto the anode during the pre‐pressing step of solid‐state anvil cell assembly is another approach for mechanical prelithiation.^[^ ^21^, ^25^ ^]^ These direct pressure‐induced prelithiation strategies can be simpler and more effective compared to the chemical and electrochemical methods. However, most reported works on mechanical prelithiation require high applied stack pressures (over 200 MPa) to accelerate the lithiation reactions,^[^ ^20^, ^21^, ^23^, ^24^, ^25^, ^26^ ^]^ or they cannot be scaled for continuous processing and are thus incompatible with roll‐to‐roll battery manufacturing. Therefore, prelithiation strategies with precise control over prelithiation extent and industrial compatibility are needed. In addition to these scalability and practicality concerns, the fundamental nature of solid‐state lithiation reactions for alloy anodes with multi‐component and multi‐phase microstructures is not well understood. Investigation of the mechanical lithiation mechanisms for alloy anodes with multi‐phase microstructures is needed to develop universal mechanical prelithiation methods.

Here, we introduce a general and scalable prelithiation method via reaction of foil and slurry‐cast alloy anodes with Li metal using warm roll‐pressing. With this method, prelithiated Si, Al, and Sn electrodes were fabricated, and the roll‐pressing‐based prelithiation process results in uniform lithiated layers with controllable thickness. The prelithiated Si particulate electrodes show a monolithic Li*_x_* Si alloy layer, while the prelithiated Al and Sn foil anodes feature a bilayer structure of reacted and unreacted regions. The dense Li alloy layer contains excess Li to enhance cycling behavior, while the underlying unreacted metal layer ensures the structural integrity of the electrode. We further investigated prelithiation behavior and microstructural evolution of multi‐phase foil‐type alloy anodes. The performance of these prelithiated electrodes was evaluated in symmetric, half, and full cells. All electrodes exhibited improved ICE, cycling capacity, and stability with prelithiation. We demonstrated cycling of prelithiated multi‐phase Al‐based foil anodes for over 400 cycles at a stack pressure of 2 MPa. Our prelithiation method via warm roll‐pressing enables improved performance of a wide variety of alloy anode materials and is compatible with conventional roll‐to‐roll battery manufacturing processes.

## 2 Mechanical Prelithiation of Alloy Anodes

**Figure 1:**
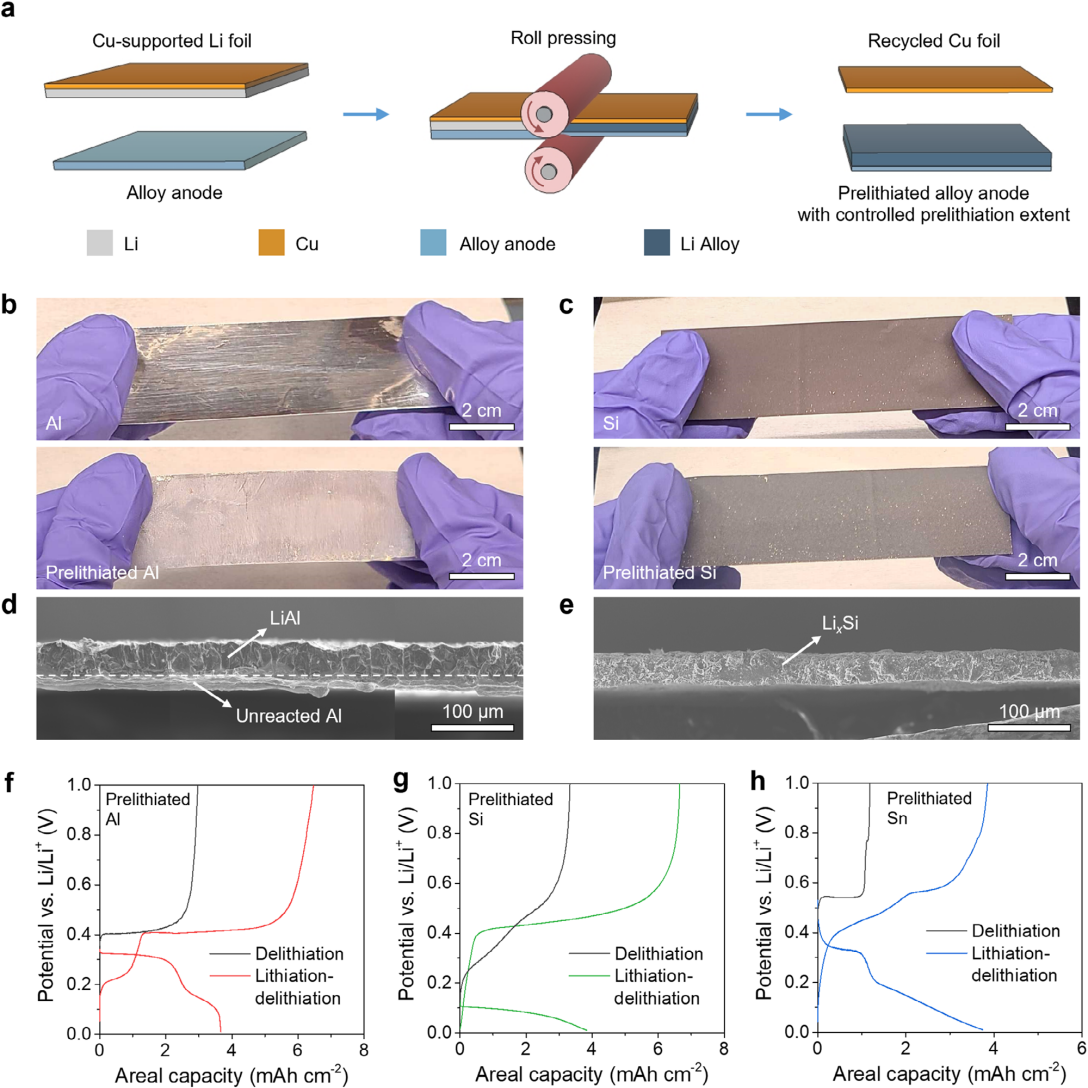
Prelithiation of film‐type alloy anodes by roll‐pressing. a) Schematic illustration of the prelithiation process. b) Photographs of an Al foil electrode before and after prelithiation. c) Photographs of an SSE‐free Si particulate electrode before and after prelithiation. d) Cross‐sectional SEM image of the prelithiated Al foil electrode (this image contains three separate images stitched together). e) Cross‐sectional SEM image of the prelithiated Si electrode. f–h) Half‐cell tests of the prelithiated alloy anodes using Li_6_PS_5_Cl SSE and Li counter electrodes. (f) Al, (g) Si, (h) Sn. The prelithiation extent was 50%. The current density was 0.1 mA cm^−2^ and the stack pressure was 5 MPa. The cutoff voltages for lithiation and delithiation were 0.01 and 1.0 V versus Li/Li^+^, respectively.

**Figure** **1**a shows a schematic illustration of the roll‐pressing‐based prelithiation process for foil‐type alloy anodes. In this process, the alloy electrode is roll pressed in contact with a Li metal foil with controlled thickness on a Cu backing foil. The thickness of the Li metal is determined by the desired extent of prelithiation, and it is maintained to be thinner than that needed for full lithiation of the electrode. The roll‐pressing temperature is 150 °C, which is below the melting point of Li but sufficiently accelerates the solid‐state lithiation reaction. During roll‐pressing with the Cu‐supported Li foil, the alloy electrode spontaneously reacts with Li metal to form Li alloy phases. A dense Li alloy layer forms at the Li‐contacting surface, while the backside of the electrode foil remains unreacted and acts as the current collector to ensure the structural integrity of the prelithiated electrode. After the reaction of the electrode with Li, the original Cu foil that supported the Li remains behind and can be reused by adding more Li. This method is suitable for various foil‐type alloy anode materials that form intermetallic phases or solid solutions with Li, including Al, Sn, Ag, and Mg foils.

We selected Al foil as a representative Li alloy material to demonstrate this method. The thickness of the Al foil was 30 µm, corresponding to a theoretical areal capacity of ≈8 mAh cm^−2^ for the LiAl phase. The Al foil was roll‐pressed with Li metal having a 20‐µm thickness on Cu foil backing, resulting in lithiation of the Al. Photographs of a typical Al foil before and after prelithiation (Figure 1b) demonstrate the uniformity of the roll‐to‐roll prelithiation method across ≈10 cm.

This method also works for slurry‐cast particulate alloy anodes such as Si. We first fabricated single‐sided SSE‐free Si electrodes by slurry casting on Cu current collectors. The electrodes were comprised of 99.9% Si micropowder and 0.1% binder, and they had an initial porosity of ≈30% after casting and drying. Figure S1 (Supporting Information) shows a cross‐sectional focused‐ion beam (FIB) scanning electron microscope (SEM) image of the pristine Si electrode. The mass loading of Si in the cast electrode was 2.3 mg cm^−2^, corresponding to a theoretical areal capacity of ≈8 mAh cm^−2^, similar to the Al foil electrodes. As with the prelithiation of foil‐type alloy anodes, the Si electrode films were directly roll‐pressed with Cu‐supported Li foils, resulting in a reaction to form a dense Li*_x_* Si alloy layer on the Cu current collector. Figure 1c shows photographs of the pristine and prelithiated Si electrodes, which show a subtle color difference, demonstrating uniformity of the prelithiation process. Figure S2 (Supporting Information) shows photographs of the pristine Cu‐supported Li foil and the ready‐to‐recycle Cu foil after roll pressing with the Si electrode. Most of the Li metal was reacted with the alloy anode during the mechanical prelithiation. X‐ray photoelectron spectroscopy (XPS) characterization of various prelithiated electrodes (Figure S3, Supporting Information) confirms that there is no trace of Cu at the surface of the lithiated alloy electrodes.

The SEM images in Figure 1d,e show cross‐sections of the prelithiated Al and Si electrodes. Li metal foils with a thickness of 20 µm were used for the prelithiation, corresponding to a capacity of 4 mAh cm^−2^ and a prelithiation extent of 50% for the two samples. With 50% prelithiation, the Al foil has a two‐layer structure with the LiAl alloy on top and unreacted Al at the bottom, while the Si electrode shows a monolithic Li*_x_* Si alloy layer. The prelithiation extent can be controlled by adjusting the Li metal thickness. Figure S4 (Supporting Information) shows cross‐sectional SEM images of Al and Si electrodes with 25% and 75% extent of prelithiation. The thickness of the Li alloy layers is dependent on the extent of prelithiation. In addition to Al and Si electrodes, we further fabricated prelithiated Sn foil electrodes using the roll‐pressing method (Figure S5, Supporting Information), and they featured a bilayer structure similar to prelithiated Al foils.

This controlled prelithiation can help mitigate the negative effects of Li trapping within alloy anodes for SSBs in the first lithiation/delithiation cycle. We examined the electrochemical behavior of these prelithiated electrodes in half‐cells with argyrodite Li_6_PS_5_Cl SSE and Li metal counter electrodes. The prelithiated electrodes were electrochemically lithiated to 0.01 V and then delithiated to 1.0 V versus Li/Li^+^. Over this lithiation/delithiation cycle, both the prelithiated Al and Si electrodes show ICEs over 150% (Figure 1f,g), while the non‐prelithiated electrodes show ICE values around 70% (Figure S6a,b, Supporting Information). Sn‐based anodes are known to suffer from severe diffusional Li trapping in SSBs due to the relatively low Li diffusivities of Li*_x_* Sn.^[^ ^10^, ^27^ ^]^ Accordingly, the non‐prelithiated Sn foil anode has a low ICE of ≈50% (Figure S6c, Supporting Information). After prelithiation, the Sn electrode exhibits an ICE of 103% (Figure 1h). The prelithiated Al, Si, and Sn samples display high areal delithiation capacities of 6.47, 6.65, and 3.85 mAh cm^−2^, respectively. Figure 1f–h also shows electrochemical curves from direct electrochemical delithiation of separate prelithiated electrodes. Upon direct delithiation, the prelithiated Al and Si electrodes provide areal capacities of 2.97 and 3.33 mAh cm^−2^, respectively. The prelithiated Sn electrode shows a relatively low delithiation capacity of 1.19 mAh cm^−2^ because of more severe diffusional Li trapping. Overall, these results show that prelithiated electrodes can be further electrochemically lithiated despite the presence of the alloy, and that prelithiation increases CE of the first lithiation/delithiation cycle.

## 3 Prelithiation Behavior of Multi‐Phase Foil Electrodes

**Figure 2:**
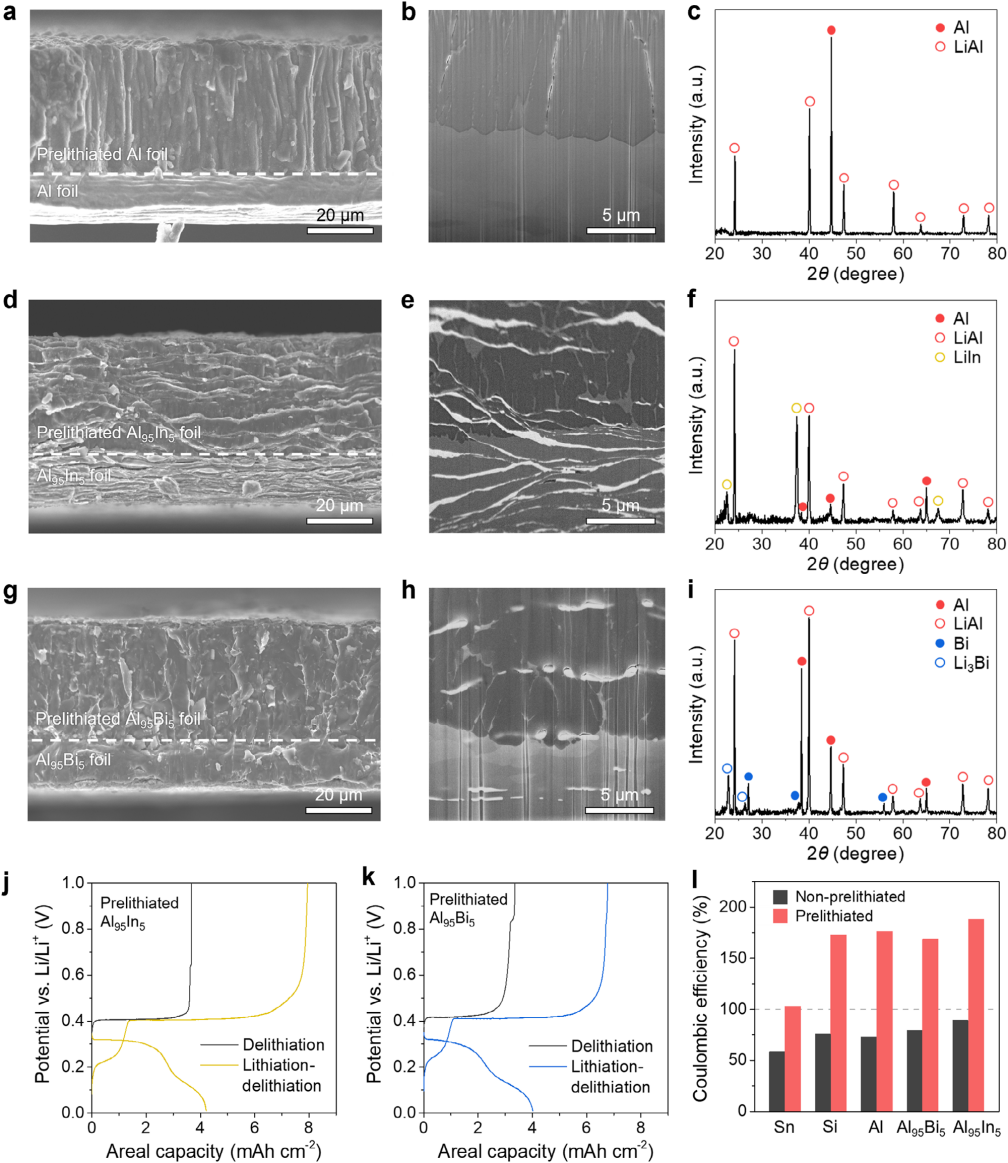
Prelithiation of pure Al and dual‐phase foil electrodes. a) Cross‐sectional SEM image of a partially prelithiated Al foil electrode. b) Cryo‐FIB‐SEM image of the prelithiation front of the partially prelithiated Al foil electrode. c) X‐ray diffraction (XRD) characterization of the partially prelithiated Al foil electrode. d) Cross‐sectional SEM image of a partially prelithiated Al‐In foil electrode (Al_95_In_5_). e) Cryo‐FIB‐SEM image of the prelithiation front of the partially prelithiated Al_95_In_5_ foil electrode. f) XRD characterization of the partially prelithiated Al_95_In_5_ foil electrode. g) Cross‐sectional SEM image of a partially prelithiated Al‐Bi foil electrode (Al_95_Bi_5_). h) Cryo‐FIB‐SEM image of the prelithiation front of the partially prelithiated Al_95_Bi_5_ foil electrode. i) XRD characterization of the partially prelithiated Al_95_Bi_5_ foil electrode. j, k) Half‐cell tests of the prelithiated dual‐phase foil electrodes using Li_6_PS_5_Cl SSE and Li counter electrodes. (j) Al_95_In_5_, (k) Al_95_Bi_5_. The current density was 0.1 mA cm^−2^ and the stack pressure was 5 MPa. The cutoff voltages for lithiation and delithiation were 0.01 and 1.0 V versus Li/Li^+^, respectively. l) Comparison of Coulombic efficiency for the first lithiation/delithiation cycle of the prelithiated and non‐prelithiated electrodes. All electrodes were prelithiated with 20 µm‐thick Cu‐supported Li foils (4.0 mAh cm^−2^ areal capacity).

We further investigated the prelithiation behavior of multi‐component foil‐based alloy anodes, which have previously been shown to exhibit enhanced cycling performance and CE due to improved Li transport and chemo‐mechanical stability.^[^ ^9^ ^]^ One component within the electrode may exhibit preferential lithiation behavior since the materials have different electrode potentials. Multi‐component Al‐based foils containing 5 at.% of a second material (In and Bi) were fabricated. In and Bi are immiscible with Al and thus form dual‐phase microstructures with Al. Dual‐phase Al_95_In_5_ and Al_95_Bi_5_ foils with a thickness of 30 µm were prelithiated using the roll‐pressing method with 20 µm‐thick Cu‐supported Li foils. **Figure** **2**a,d,g shows cross‐sectional SEM images of the prelithiated Al (Figure 2a) and multi‐component foil electrodes (Figure 2d,g) after prelithiation. All prelithiated samples again feature a bilayer structure, with the Li alloy layer on top of the unreacted foil, indicating that the Li metal spontaneously reacts to form Li alloy phases.

We characterized the microstructure as well as the distribution of the secondary phase in the multi‐component foil electrodes after prelithiation using cryo‐FIB SEM. Prelithiated pure Al shows a uniform reaction front and distinct grayscale contrast between the LiAl and pure Al (Figure 2b), with the XRD revealing both LiAl and Al present (Figure 2c). The prelithiated Al_95_In_5_ sample (Figure 2d,e) shows a 3D network of LiIn throughout the Al foil matrix, and the LiAl reaction front extends about halfway down the foil. As demonstrated in our previous work, the Al‐In foils feature an interconnected In network formed during anisotropic cold rolling.^[^ ^9^, ^28^ ^]^ During prelithiation, the In phase reacts with Li first because of its higher electrode potential compared to Al, while the LiAl phase only partially extends into the foil. XRD characterization of the prelithiated Al_95_In_5_ foil (Figure 2f) shows the presence of LiAl, LiIn, and pure Al in the sample, confirming that all In in the sample was lithiated while some Al remained.

Different from Al‐In foils, the Al‐Bi foils feature Bi particles distributed in the Al matrix; these particulate domains form due to solid‐phase precipitation during cooling. Figure 2h shows a cryo‐FIB‐SEM image of the Al_95_Bi_5_ foil after partial prelithiation. The prelithiated sample has a bilayer LiAl/Al structure, with the Bi or Li_3_Bi phases dispersed in the Al foil matrix. Figure 2i shows XRD data of the prelithiated Al_95_Bi_5_ foil, revealing the presence of LiAl, pure Al, Li_3_Bi, and pure Bi in the sample. Since the secondary Bi phase is not interconnected in the pristine foil, this prevents full reaction of the Bi phase despite the high electrode potential of Bi,^[^ ^10^ ^]^ and both Bi and Li_3_Bi are present. Thus, the chosen processing route and initial foil microstructure determine the prelithiation behavior and the final distribution of lithiated phases.

As shown in Figure 2j,k, the prelithiated multi‐phase foil anodes exhibit higher delithiation capacity and improved electrochemical behavior in SSBs compared to the prelithiated single‐phase Al foil (all foils received 4.0 mAh cm^−2^ Li during prelithiation). The secondary LiIn and Li_3_Bi phases distributed in the foil matrix can support relatively fast Li diffusion and minimize Li trapping. In particular, the interconnected 3D network of the LiIn phase in the prelithiated Al_95_In_5_ sample provides high‐diffusivity transport channels throughout the foil,^[^ ^9^ ^]^ enabling an ICE of 188.5% in the first cycle with an areal delithiation capacity of 7.95 mAh cm^−2^ after full lithiation. The prelithiated Al_95_Bi_5_ sample displays an ICE of 168.7% with an areal delithiation capacity of 6.77 mAh cm^−2^. Upon direct delithiation, all the prelithiated multi‐phase electrodes provide high areal capacities above 3.0 mAh cm^−2^ (Figure 2j,k), which is higher than the pure Al foil cases (Figure 1f). The delithiation voltage curves in Figure 2j,k show slight plateaus at ≈0.64 and ≈0.84 V versus Li/Li^+^, which correspond to the delithiation of the LiIn and Li_3_Bi phases in the multi‐phase foil electrodes, respectively. Figure 2l and Table S1 (Supporting Information) compare the ICE values of all the prelithiated and non‐prelithiated electrodes. With prelithiation, the alloy anodes exhibit significantly increased CE values of the first lithiation/delithiation cycle.

## 4 Electrochemical Behavior of Prelithiated Electrodes

**Figure 3:**
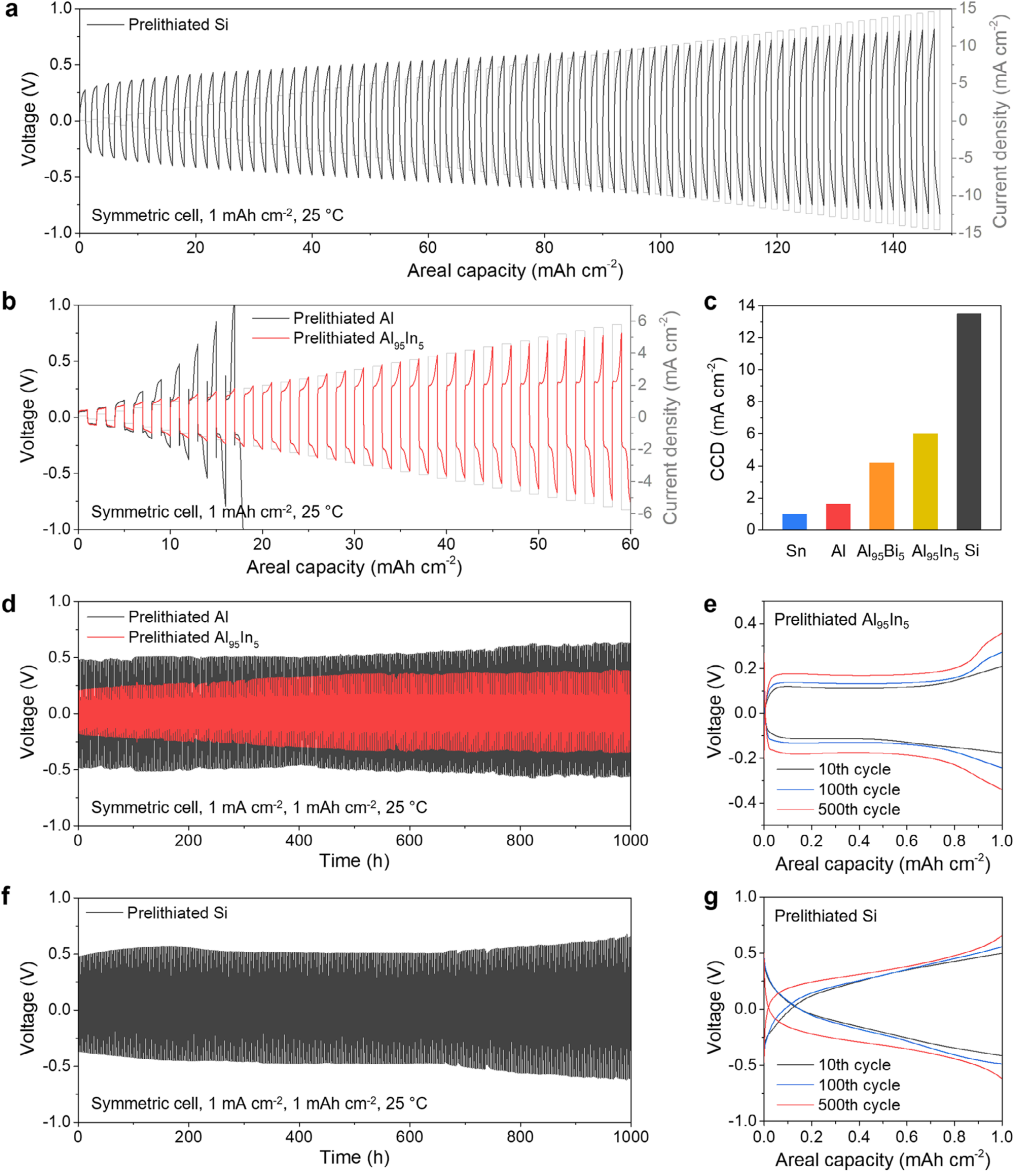
Electrochemical behavior of prelithiated electrodes. a) CCD test of prelithiated Si electrodes in a symmetric cell with Li_6_PS_5_Cl SSE under a stack pressure of 50 MPa. b) CCD tests of prelithiated Al and Al_95_In_5_ electrodes in symmetric cells with Li_6_PS_5_Cl SSE and a stack pressure of 50 MPa. c) Comparison of CCD values of the prelithiated electrodes, all under a stack pressure of 50 MPa. d) Cycling performance of prelithiated Al and Al_95_In_5_ foil electrodes in symmetric cells with 1 mA cm^−2^ current density and 50 MPa stack pressure. e) Voltage curves from the cycling test in (d). f) Cycling performance of prelithiated Si electrodes in a symmetric cell with 1 mA cm^−2^ current density and 50 MPa stack pressure. g) Voltage curves from the cycling test in (f).

The prelithiated electrodes were assembled in symmetric cells with Li_6_PS_5_Cl SSE to determine their critical current density (CCD). The CCD is a somewhat ill‐defined but widely used metric for assessing the maximum current density that a cell can endure before experiencing short‐circuiting due to Li filament penetration.^[^ ^29^ ^]^ CCD tests were carried out with a current density ramp of 0.2 mA cm^−2^ per step and a capacity of 1.0 mAh cm^−2^ during each half‐cycle. Representative CCD tests on cells with prelithiated Si, Al, and Al_95_In_5_ electrodes are shown in **Figure** **3**a,b, and additional experimental results from other samples are shown in Figure S7 (Supporting Information). At low current densities, the prelithiated Si electrode (Figure 3a) shows a higher overpotential compared to the Al‐based samples (Figure 3b), which is likely due to the higher voltage hysteresis of Si (Figure 1f,g). With higher current densities, the cells exhibit higher polarization. However, the polarization of the prelithiated Si electrode increases with a smaller slope than the prelithiated Al electrodes, indicating faster reaction and/or diffusion kinetics. The single‐phase lithiation/delithiation reactions of amorphous Li*_x_* Si do not feature distinct reaction fronts like the Al/LiAl interface. After delithiation, the pure Al phase formed at the electrode/SSE interface could cause the voltage polarization at high current densities due to its relatively low Li diffusivity. Figure S8 (Supporting Information) shows the repeated CCD tests for the prelithiated Si electrode. The average CCD value is 13.5 ± 0.8 mA cm^−2^, which is, to our knowledge, the highest in literature for Si‐based electrodes in SSBs.^[^ ^11^, ^30^ ^]^

As shown in Figure 3b, the prelithiated Al_95_In_5_ electrode shows a lower polarization and higher CCD than the prelithiated Al electrode, providing evidence that the distributed LiIn phase within the Al foil matrix enhances Li transport and rate behavior. Figure 3c displays the CCD values of all the prelithiated electrodes. Compared to prelithiated Al foil (CCD of 1.6 mA cm^−2^), the prelithiated multi‐phase foil electrodes have higher CCD values (4.2 mA cm^−2^ for Al_95_Bi_5_ and 5.8 mA cm^−2^ for Al_95_In_5_). These data show that microstructural design and the addition of a secondary phase within Al‐based materials are effective approaches for improved rate behavior and performance. The prelithiated Sn electrode has a lower CCD of 1.2 mA cm^−2^ due to the relatively poor lithiation/delithiation kinetics of Li‐Sn phases.

We next evaluated the long‐term cycling capability of the prelithiated electrodes in symmetric cells. Figure 3d,f shows the galvanostatic voltage profiles of prelithiated Al, Al_95_In_5_, and Si electrodes cycled at 1 mA cm^−2^ with a half‐cycle capacity of 1 mAh cm^−2^. The prelithiated electrodes exhibit stable cycling for 500 cycles (1000 h) without any short‐circuiting, demonstrating excellent interfacial stability of the Li alloys with the SSE. Electrochemical impedance spectroscopy (EIS) measurement results in Figure S9 (Supporting Information) show that the impedance of the symmetric cells increased slightly after 500 cycles, indicating stable interfaces between the prelithiated alloy anodes and Li_6_PS_5_Cl. As shown by the voltage curves in Figure 3e,g, the overpotentials of these cells have a slight increase of 100–150 mV after 500 cycles. Compared to the prelithiated Al electrode, the multi‐phase Al_95_In_5_ electrode has a ≈200 mV lower overpotential during cycling.

**Figure 4:**
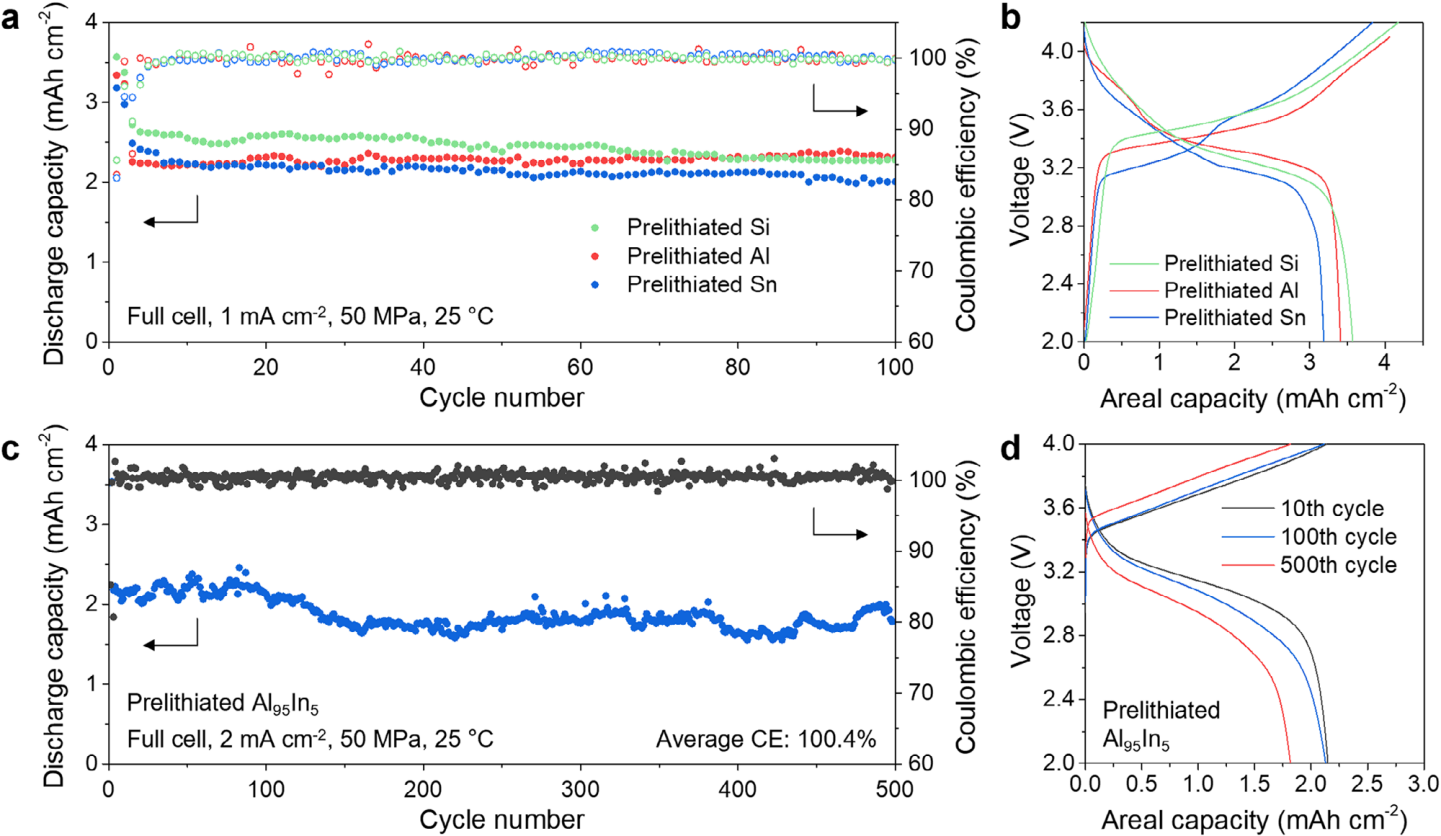
Electrochemical performance of prelithiated electrodes in full cells. a) Galvanostatic cycling of prelithiated Si, Al, and Sn electrodes in full cells with LiNi_0.6_Co_0.2_Mn_0.2_O_2_ cathodes. The current density was 0.2 mA cm^−2^ for the first two cycles and 1.0 mA cm^−2^ for subsequent cycles. The cathode loading was 4.0 mAh cm^−2^. The stack pressure was 50 MPa. b) First‐cycle voltage curves from the cycling tests. c) Galvanostatic cycling of the prelithiated Al_95_In_5_ foil electrode in a full cell with LiNi_0.6_Co_0.2_Mn_0.2_O_2_ cathode. The current density was 0.2 mA cm^−2^ for the first two cycles and 2.0 mA cm^−2^ for subsequent cycles. The cathode loading was 4.0 mAh cm^−2^. The stack pressure was 50 MPa. d) Voltage curves from the cycling tests in (c).

In full cells, the incorporation of Li directly within the electrodes before battery assembly allows for excess Li to be present in the cell, which is helpful for mitigating any loss of Li due to side reactions during charge/discharge cycling. **Figure** **4** shows galvanostatic data from SSB cells with the prelithiated alloy anodes, Li_6_PS_5_Cl SSE separators, and LiNi_0.6_Mn_0.2_Co_0.2_O_2_ (NMC622) cathodes. A current density of 0.2 mA cm^−2^ was used for the first two cycles, with 1 or 2 mA cm^−2^ used for subsequent cycles. A high stack pressure of 50 MPa was used to minimize any effects of interfacial contact loss for this analysis. As shown in Figure 4a, the cells with the prelithiated Si, Al, and Sn electrodes exhibit high average cycling capacity (2.47 mAh cm^−2^ for Si, 2.31 mAh cm^−2^ for Al, and 2.16 mAh cm^−2^ for Sn) and good stability over 100 cycles. Cells with prelithiated Si, Al, and Sn electrodes were tested in triplicate, with representative first‐cycle voltage curves shown in Figures 4b and S10 (Supporting Information) showing all curves. The average ICE values are 84.2% ± 0.6% for prelithiated Al, 85.8% ± 0.5% for prelithiated Si, and 84.5% ± 1.2% for prelithiated Sn. Compared to the ICE values of over 100% in half cells, the ICE of full cells is likely limited by the NMC cathode,^[^ ^31^, ^32^ ^]^ which has an ICE around 85%. The differential capacity (dQ/dV) curves in Figure S11 (Supporting Information) show the main peaks from lithiation/delithiation of the three samples. For comparison, we tested non‐prelithiated Si, Al, and Sn electrodes under the same conditions. The results from these cells in Figure S12 (Supporting Information) display lower capacities (<2.0 mAh cm^−2^) and ICE values of 50–75%, demonstrating that the prelithiation clearly increases the ICE, cycling capacity, and cyclability of the alloy anodes in full cells. We further tested the prelithiated Si and Al_95_In_5_ electrodes in full cells with LiCoO_2_ (LCO) cathodes, which feature improved ICE compared to NMC cathodes. As shown by the galvanostatic cycling results in Figure S13 (Supporting Information), the ICE of the LCO cells increased to 88% for the prelithiated Si electrode and 91% for the prelithiated Al_95_In_5_ electrode, confirming that the cathode is limiting ICE in these cells.

The prelithiated multi‐phase electrodes also show promising cycling performance in SSB full cells. The cell with the prelithiated Al_95_In_5_ foil (Figure 4c) shows excellent stability over 500 cycles, with an areal capacity of ≈2.0 mAh cm^−2^ at a higher current density of 2 mA cm^−2^. The corresponding galvanostatic voltage curves on the tenth, 100th, and 500th cycles are shown in Figure 4d. Figure S14 (Supporting Information) shows galvanostatic full‐cell cycling test results for a prelithiated Al_95_Bi_5_ foil electrode. At a current density of 1 mA cm^−2^, the prelithiated Al_95_Bi_5_ foil also exhibits high capacity (≈2.8 mAh cm^−2^) and stable cycling over 100 cycles. Table S2 (Supporting Information) compares the cycling results in Figures 4 and S14 (Supporting Information) to other recent demonstrations of alloy anode‐based SSBs.

## 5 Low‐Stack‐Pressure Performance of Prelithiated Multi‐Phase Foil Anodes

For practical applications of SSBs, low operating stack pressures (<2 MPa) are required because of the significant size and mass of stack plates needed for higher pressure application.^[^ ^5^, ^33^, ^34^ ^]^ However, high stack pressures are widely used for alloy anodes in SSBs in the research literature because of the higher yield strength and Young's modulus of Li alloys compared to pure Li metal.^[^ ^2^, ^11^, ^35^ ^]^ The applied stack pressure affects the structural evolution of the alloy anode and its contact with the SSE during cycling. Low stack pressures can lead to short‐circuiting and faster capacity decay due to contact loss at the interface during long‐term cycling. To achieve improved performance of the prelithiated alloy anodes at lower stack pressures, we used the strategy developed in our previous work,^[^ ^14^ ^]^ which involves adding a Li‐conducting interfacial material at the anode/SSE interface. The interfacial material used here is a 2‐µm In metal layer, which reacts to form LiIn and remains dense at the interface, thus maintaining stable interfacial contact with the SSE during charge‐discharge cycling at low stack pressures.^[^ ^14^ ^]^

**Figure 5:**
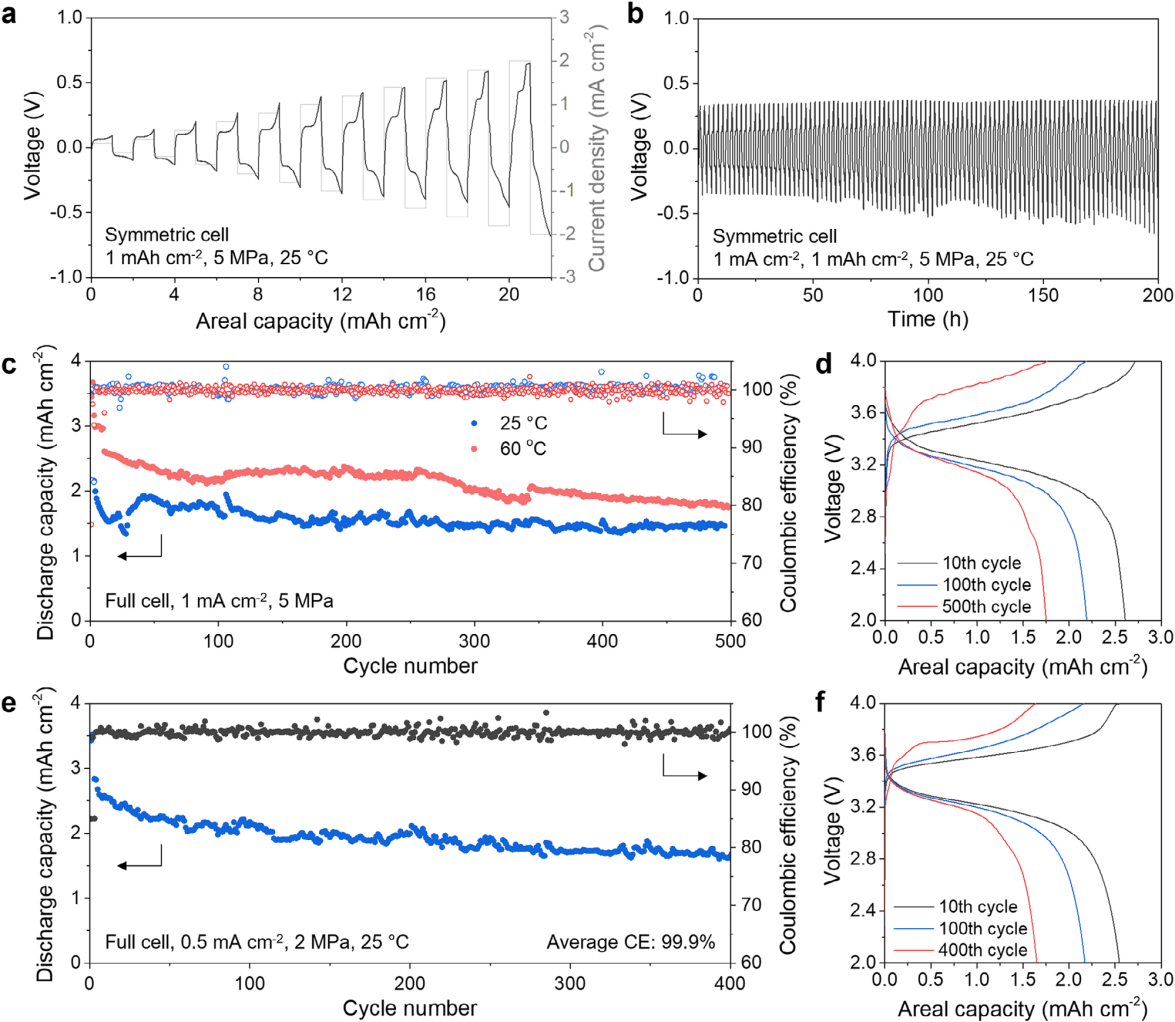
Low‐stack‐pressure electrochemical behavior of prelithiated multi‐phase Al‐based foil electrodes. a) CCD test of prelithiated Al_95_In_5_ foil electrodes with an In interfacial layer coating in a symmetric cell under 5 MPa. b) Cycling performance of prelithiated Al_95_In_5_ foil electrodes with In interfacial layer in a symmetric cell with 1 mA cm^−2^ current density under 5 MPa. c) Cycling performance of a prelithiated Al_95_In_5_ foil anode with In interfacial layer coating in a full cell with LiNi_0.6_Co_0.2_Mn_0.2_O_2_ cathode at 25 and 60 °C. The current density was 0.2 mA cm^−2^ for the first two cycles and 1.0 mA cm^−2^ for subsequent cycles. The cathode loading was 4.0 mAh cm^−2^ and the stack pressure was 5 MPa. d) Voltage curves from the cycling test at 60 °C in (c). e) Cycling performance of a prelithiated Al_95_In_5_ foil anode with In interfacial layer in a full cell with LiNi_0.6_Co_0.2_Mn_0.2_O_2_ cathode at 25 °C. The current density was 0.2 mA cm^−2^ for the first two cycles and 0.5 mA cm^−2^ for subsequent cycles. The cathode loading was 4 mAh cm^−2^ and the stack pressure was 2 MPa. f. Voltage curves from the cycling test in (e).

**Figure** **5**a,b shows the low‐stack‐pressure testing results from symmetric cells with prelithiated Al_95_In_5_ foil electrodes with the In interfacial layer. The CCD of the coated and prelithiated Al_95_In_5_ electrodes is 2 mA cm^−2^ at 5 MPa (Figure 5a), while the uncoated sample has a CCD less than 1 mA cm^−2^ under the same testing conditions (Figure S15, Supporting Information). The symmetric cell cycling test in Figure 5b demonstrates good stability for 100 cycles.

The interfacial layer is also effective for enhancing the performance of the prelithiated electrodes in full cells at low stack pressures. We carried out cycling tests of NMC622 cells containing prelithiated Al_95_In_5_ electrodes with the In interfacial layer at low stack pressures at 25 and 60 °C. As shown by Figure 5c,d, the cells tested at 5 MPa exhibited stable cycling over 500 cycles and high areal capacities (≈2.2 mAh cm^−2^ at 60 °C and ≈1.5 mAh cm^−2^ at 25 °C). We further reduced the applied stack pressure to 2 MPa. At room temperature and 0.5 mA cm^−2^, a cell with the In‐coated and prelithiated Al_95_In_5_ foil showed good stability for 400 cycles, with the areal capacity slowly decaying from 2.5 to 1.8 mAh cm^−2^ (Figure 5e,f).

## 6 Conclusion

This work introduces a general and scalable prelithiation method for alloy anodes via direct solid‐state lithiation using roll‐pressing with a Li film on a Cu support foil. Compared with other prelithiation methods, this technique offers advantages including high‐area reaction processes, lower complexity, and better roll‐to‐roll adaptability. We demonstrate the prelithiation of single‐component Si, Al, and Sn electrodes as well as multi‐component Al_95_In_5_ and Al_95_Bi_5_ foil anodes with this roll‐pressing method. The prelithiated electrodes show significantly enhanced ICE and cycling capability. With a Li‐transporting interfacial layer that mitigates contact loss, the prelithiated foil anodes show high capacity and stable cycling at low stack pressure (2 MPa) in SSBs, approaching practical operating conditions. Our work demonstrates a universal and scalable methodology for prelithiation of alloy‐type anodes for improved performance in SSBs.

## 7 Experimental Section

### Anode Preparation

Pure Al foils (>99.999%, 30 µm, Laurand Associates) were used as received. Sn foils were made by rolling Sn (>99.99%, Kurt J. Lesker) ingots with an electric roller to a thickness of 15 µm. The Si particulate electrodes were prepared using micro silicon particles (Alfa Aesar), 0.1 wt.% polyvinylidene difluoride (PVDF) binder, and n‐methyl‐2‐pyrrolidone (Sigma–Aldrich) solvent. A slurry was prepared and cast on a Cu foil current collector using a doctor blade. The cast electrode was dried under vacuum at 60 °C overnight. For the fabrication of multi‐phase Al foils, stoichiometric amounts of Al and other elements were placed in a crucible and melted together, followed by natural cooling. The ingots were then rolled via an electric roller to a thickness of 30 µm. The rolling was performed along one direction until the foil thickness was reduced to the desired thickness. The Al foil samples with In interfacial layer coating were prepared by immersing Al foils in an InCl_3_ solution, during which a 2‐µm In layer was deposited via a galvanic replacement reaction. For the roll‐pressing‐based prelithiation, the electrode material and Li foils were roll‐pressed together via an electric roller (MTI Corporation) at 150 °C. The prelithiation degree was controlled by adjusting the Li metal thickness (20 µm Li corresponds to 50% prelithiation extent). The gap between the rollers was ≈50 µm. The speed of the roller was ≈10 cm min^−1^.

### Cathode Preparation

The cathode was a composite mixture of LiNi_0.6_Mn_0.2_Co_0.2_O_2_ (NMC622) active material, Li_6_PS_5_Cl solid electrolyte, and vapor‐grown carbon fiber (VGCF). LiNb_0.5_Ta_0.5_O_3_ was coated on the active cathode material to prevent side reactions with the sulfide electrolyte. Stoichiometric amounts of niobium ethoxide, tantalum butoxide, and lithium acetate were dissolved in dry ethanol. NMC622 powder was mixed into this solution via sonication, and then the solvent was evaporated in a vacuum oven. The dried powder was then annealed at 450 °C. The composition of the cathode was 70 wt.% coated NMC622, 27.5 wt.% Li_6_PS_5_Cl, and 2.5 wt.% VGCF. The mixture was dry ball milled in a ZrO_2_ jar.

### Half Cell Assembly

The solid‐state separator layer was fabricated by uniaxially pressing Li_6_PS_5_Cl powders at 250 MPa inside the anvil cell. Then, the working electrode was added and pressed at 375 MPa. A Li metal foil was added to the counter electrode side and pressed at 50 MPa. Half cells were tested under a stack pressure of 5 MPa.

### Full Cell Assembly

The solid‐state separator layer was fabricated by pressing Li_6_PS_5_Cl powders at 125 MPa inside the anvil cell. Then, cathode composite powders were added and pressed at 250 MPa. The foil anode was added to the opposite side of the cell before pressing the full cell to 375 MPa. For all full cell tests in this work, the cathode loading was 4 mAh cm^−2^. Full cells were tested under a stack pressure of 2, 5, or 50 MPa at 25 or 60 °C.

### Materials Characterization

For cross‐sectional SEM, the prelithiated foils were cut with a scalpel within an Ar glove box. Images were collected on a Hitachi SU8230 SEM using an accelerating voltage of 10 kV. Cryogenic FIB‐SEM images were collected on a Thermo Fisher Helios 5CX instrument with a Quorum cryo‐stage. FIB milling of the electrodes used 30 keV Ga^+^ ions at 9.4 nA, and polishing was performed at 2.5 nA. For XRD samples, the foils were placed between Kapton tape and a glass slide within a glove box. XRD data was collected on a Panalytical Empyrean instrument, with scans from 20° to 90° with 45 keV voltage, 40 mA current, and copper K‐α radiation.

XPS characterization was carried out using a Thermo K‐Alpha XPS instrument with an Al Kα source. Samples were prepared in an Ar‐filled glovebox and transferred to the XPS instrument with a vacuum transfer holder without exposure to ambient air. The XPS spectra were collected using a 400 µm spot size with a base pressure less than 2.5 × 10^−7^ mbar. Surface charging was compensated with a flood gun.

## Conflict of Interest

Some of the authors are inventors on patent applications related to alloy anode materials for Li batteries.

## Author Contributions

C.W. and M.T.M. performed conceptualization. C.W. performed methodology. C.W., W.J.J., D.L.N., H.S., and Y.L. performed investigation. C.W. and M.T.M. performed formal analysis. C.W. and M.T.M. performed validation. C.W. wrote the original draft. C.W. and M.T.M. wrote reviewed and edited the final manuscript. C.W. and M.T.M. performed visualization. M.T.M. performed project administration. M.T.M. performed resources. M.T.M. performed supervision. M.T.M. acquired funding acquisition.

## Supporting information



Supporting Information

## Data Availability

The data that support the findings of this study are available in the supplementary material of this article.
