# Supplementary material for: Prelithiation of Alloy Anodes via Roll Pressing for Solid‐State Batteries
Source: Adv Mater. 2025 Aug 23;37(44):e08973. doi: 10.1002/adma.202508973 (PMC12592915; doi:10.1002/adma.202508973)
Supplement: Supplementary file 1 — Supporting Information [file ADMA-37-e08973-s001.docx]

*Supporting Information*

**Prelithiation of Alloy Anodes via Roll Pressing for Solid-State Batteries**

Congcheng Wang^1^, Won Joon Jeong^2^, Douglas Lars Nelson^2^, Hari Sridhara^2^, Sophia Nicolette Anderson^2^, and Matthew T. McDowell^1,2^*

^1^George W. Woodruff School of Mechanical Engineering, Georgia Institute of Technology, Atlanta, GA, USA.

^2^School of Materials Science and Engineering, Georgia Institute of Technology, Atlanta, GA, USA.

*Corresponding Author: [mattmcdowell@gatech.edu](mailto:mattmcdowell@gatech.edu)


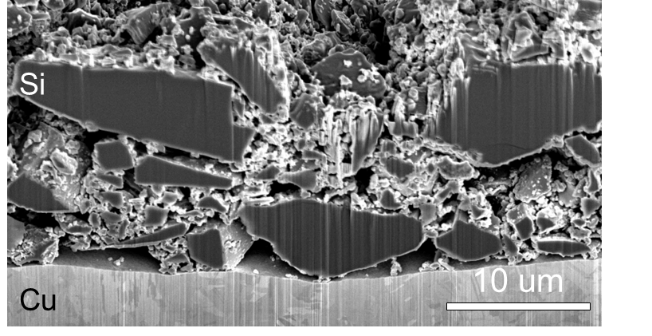


**Figure S1. Cryogenic focused ion beam scanning electron microscopy (Cryo-FIB SEM) image of a pristine particulate Si electrode.**


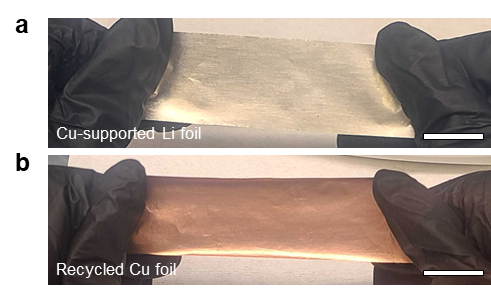


**Figure S2. Photographs of the Cu-supported Li foil** (**a**) **and Cu foil after the prelithiation process** (**b**)**.**


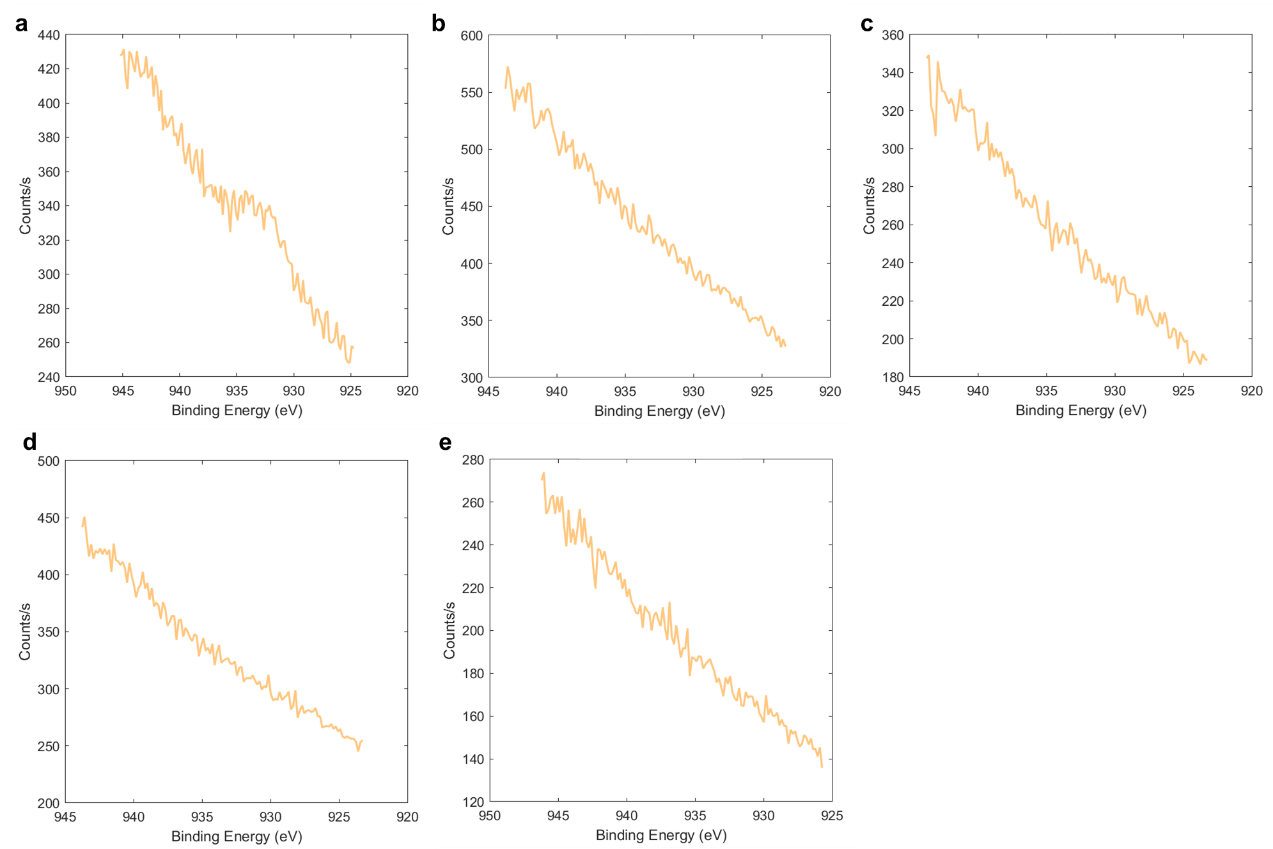


**Figure S3. X-ray photoelectron spectroscopy (XPS) Cu 2p spectra from the prelithiated electrodes. a**, prelithiated Si. **b**, prelithiated Al. **c**, prelithiated Sn. **d**, prelithiated Al_95_In_5_. (**e**), prelithiated Al_95_Bi_5_.


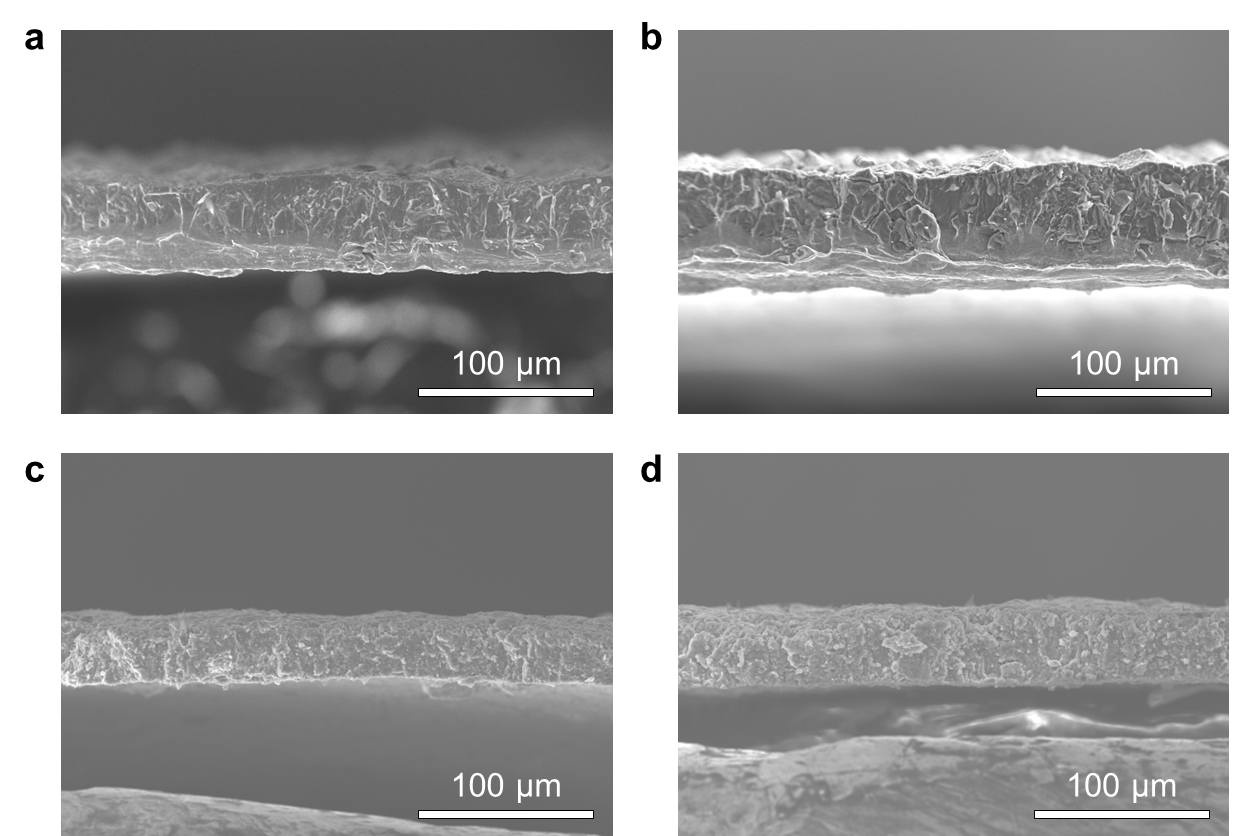


**Figure S4. Cross-sectional SEM images of prelithiated Al and Si electrodes using different thicknesses of Li. a**, Al at 25% extent of prelithiation. **b**, Al at 75% extent of prelithiation. **c**, Si at 25% extent of prelithiation. **d**, Si at 75% extent of prelithiation.


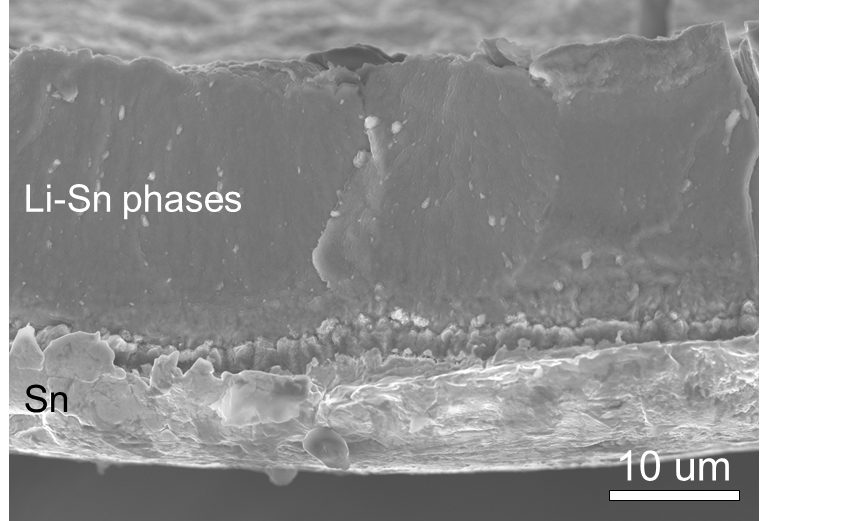


**Figure S5. Cross-sectional SEM image of the 50% prelithiated Sn foil electrode.**


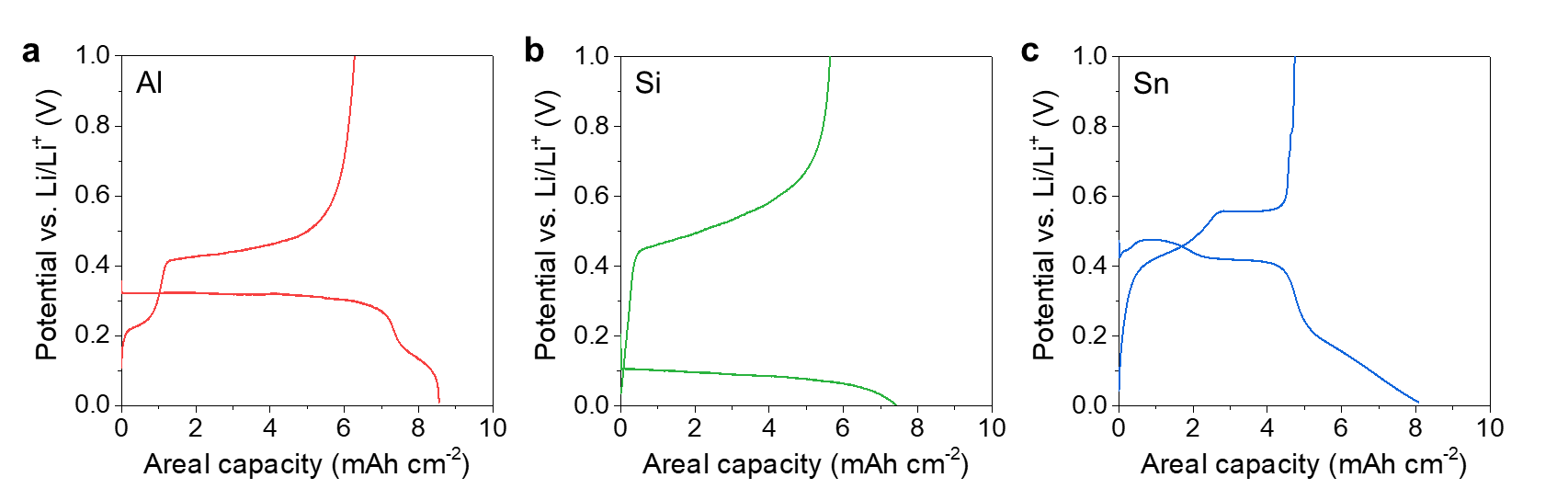


**Figure S6. Half-cell tests of pristine (****non-prelithiated) alloy electrodes.** **a**, Al. **b**, Si. **c**, Sn. The current density was 0.1 mA cm^-2^. The stack pressure was 5 MPa. The cutoff voltages for lithiation and delithiation are 0.01 V and 1.0 V vs. Li/Li^+^, respectively.

**Table S1.** Values of the lithiation/delithiation capacities and Coulombic efficiencies in Fig. 1f-h and Fig. 2j-k. The “overall CE” = [(electrochemical delithiation capacity) / (electrochemical lithiation capacity + prelithiation capacity)].

| Anode | Lithiation capacity  (mAh cm^-2^) | Delithiation capacity  (mAh cm^-2^) | CE from electrochemical lithiation/delithiation (%) | Overall CE  (%) |
| --- | --- | --- | --- | --- |
| Prelithiated Al | 3.67 | 6.47 | 176.3 | 84.4 |
| Prelithiated Si | 3.84 | 6.65 | 173.2 | 84.8 |
| Prelithiated Sn | 3.75 | 3.85 | 102.7 | 49.7 |
| Prelithiated Al_95_In_5_ | 4.22 | 7.95 | 188.4 | 96.7 |
| Prelithiated Al_95_Bi_5_ | 4.01 | 6.77 | 168.8 | 84.5 |


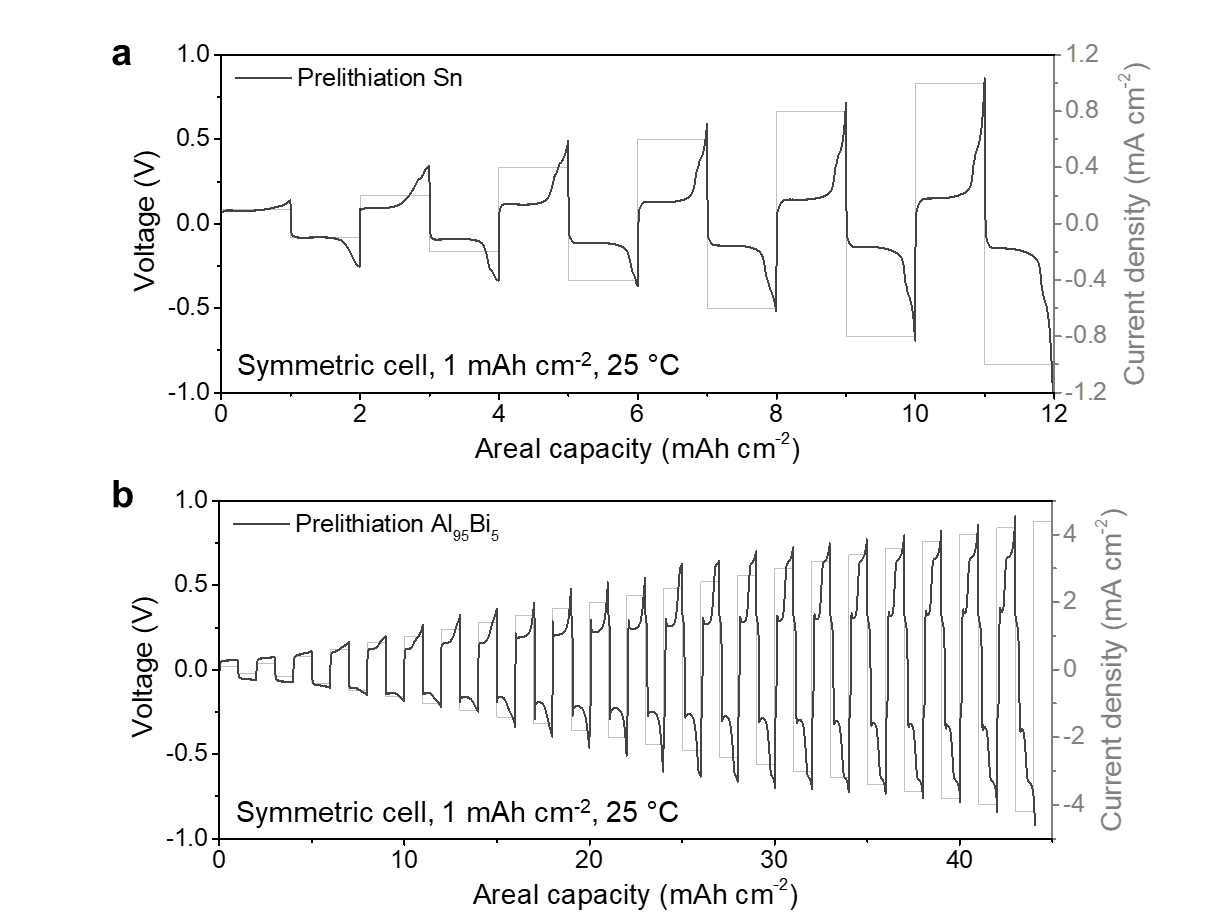


**Figure S7. Critical current density (CCD) tests of the prelithiated electrodes in symmetric cells. a**, prelithiated Sn. **b**, prelithiated Al_95_Bi_5_. The stack pressure was 50 MPa.


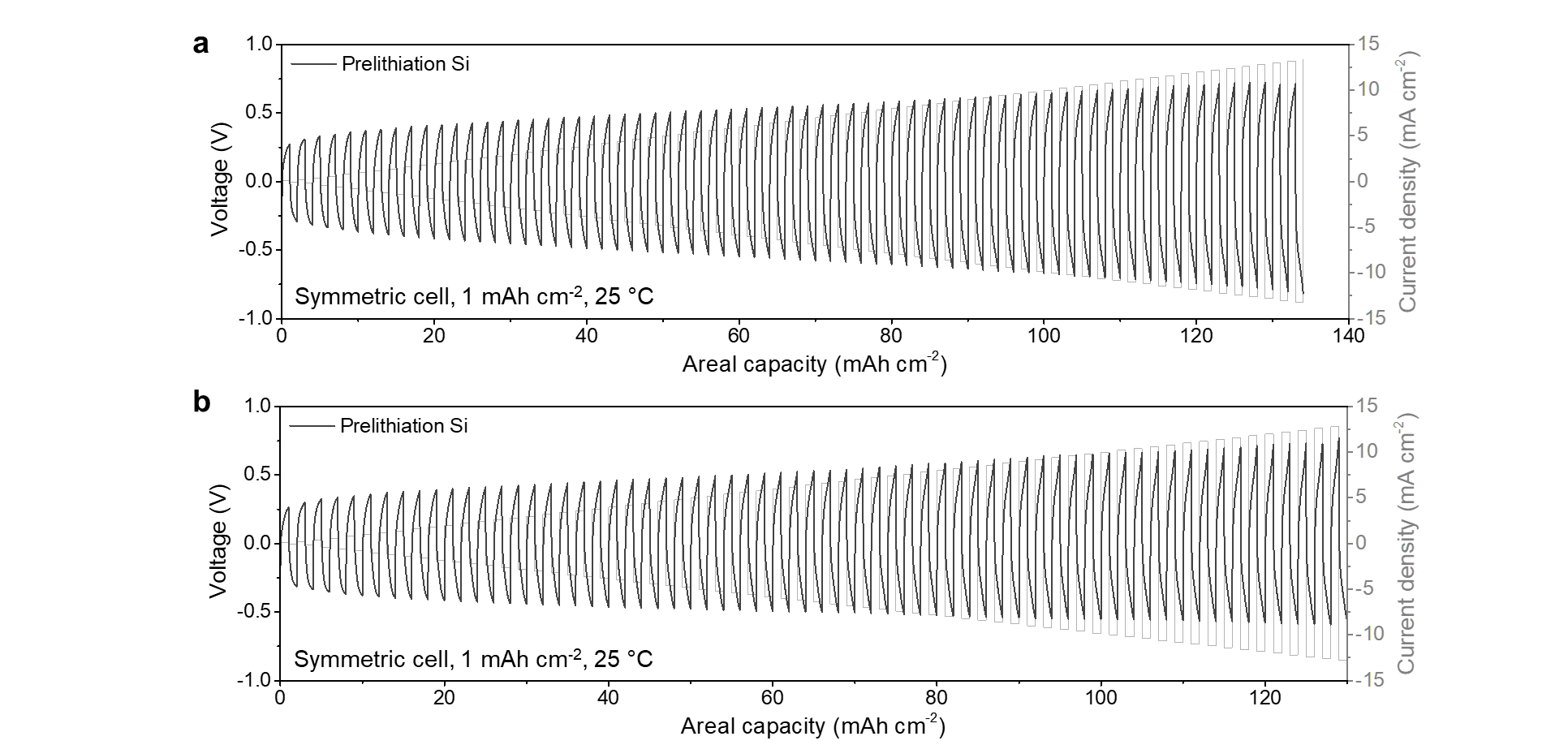


**Figure S8. Repeated CCD tests of the prelithiated Si electrodes in symmetric cells.** The stack pressure was 50 MPa.


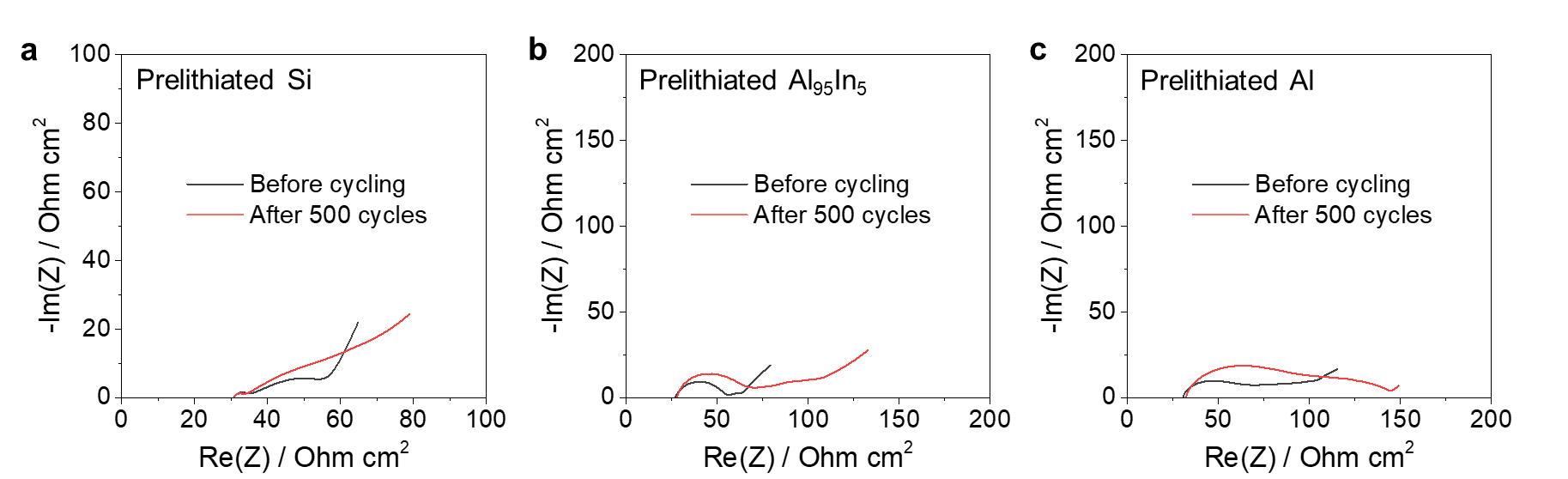


**Figure S9. Electrochemical impedance spectroscopy (EIS) measurement of the symmetric cells before/after cycling tests in Fig. 3d and f. a**, prelithiated Si. **b**, prelithiated Al_95_In_5_. **c**, prelithiated Al.


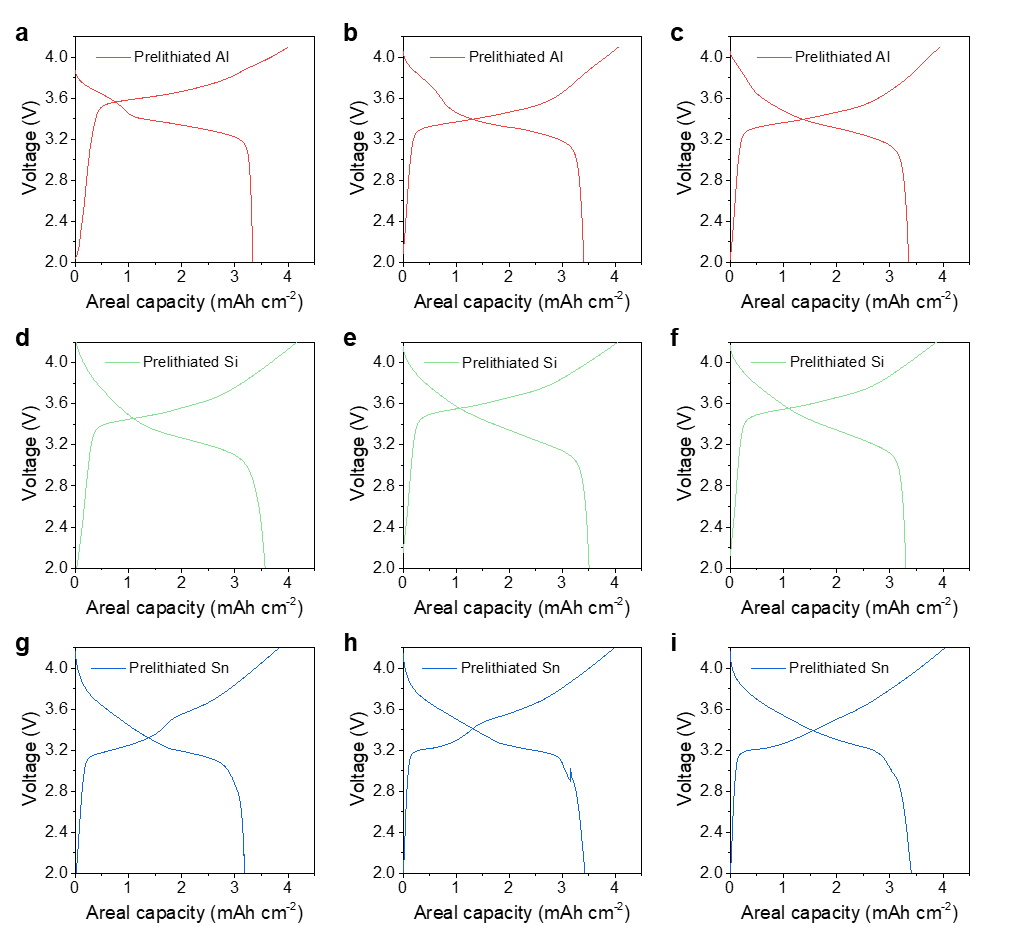


**Figure S10. First-cycle voltage curves from triplicate full cell tests of the prelithiated electrodes with NMC cathodes.** (**a**-**c**), prelithiated Al. (**d**-**f**), prelithiated Si. (**g**-**i**), prelithiated Sn.


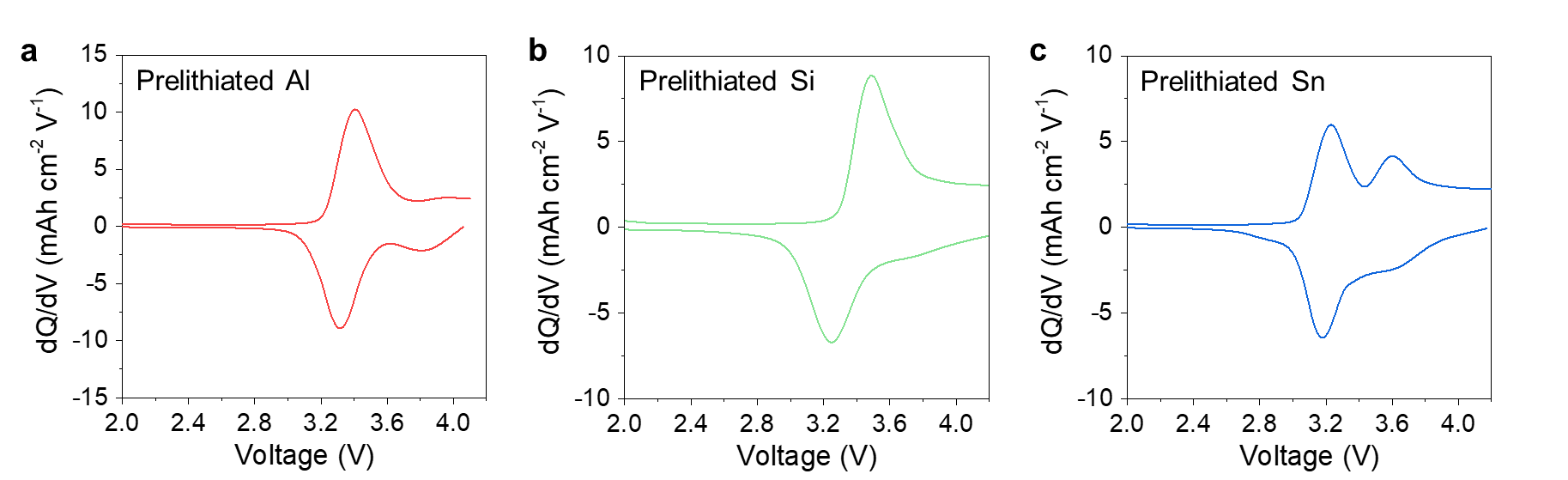


**Figure S11. Differential capacity (dQ/dV) analysis of the first cycle voltage curves from the cycling tests in Fig. 4a-b.** **a**, prelithiated Al. **b**, prelithiated Si. **c**, prelithiated Sn.


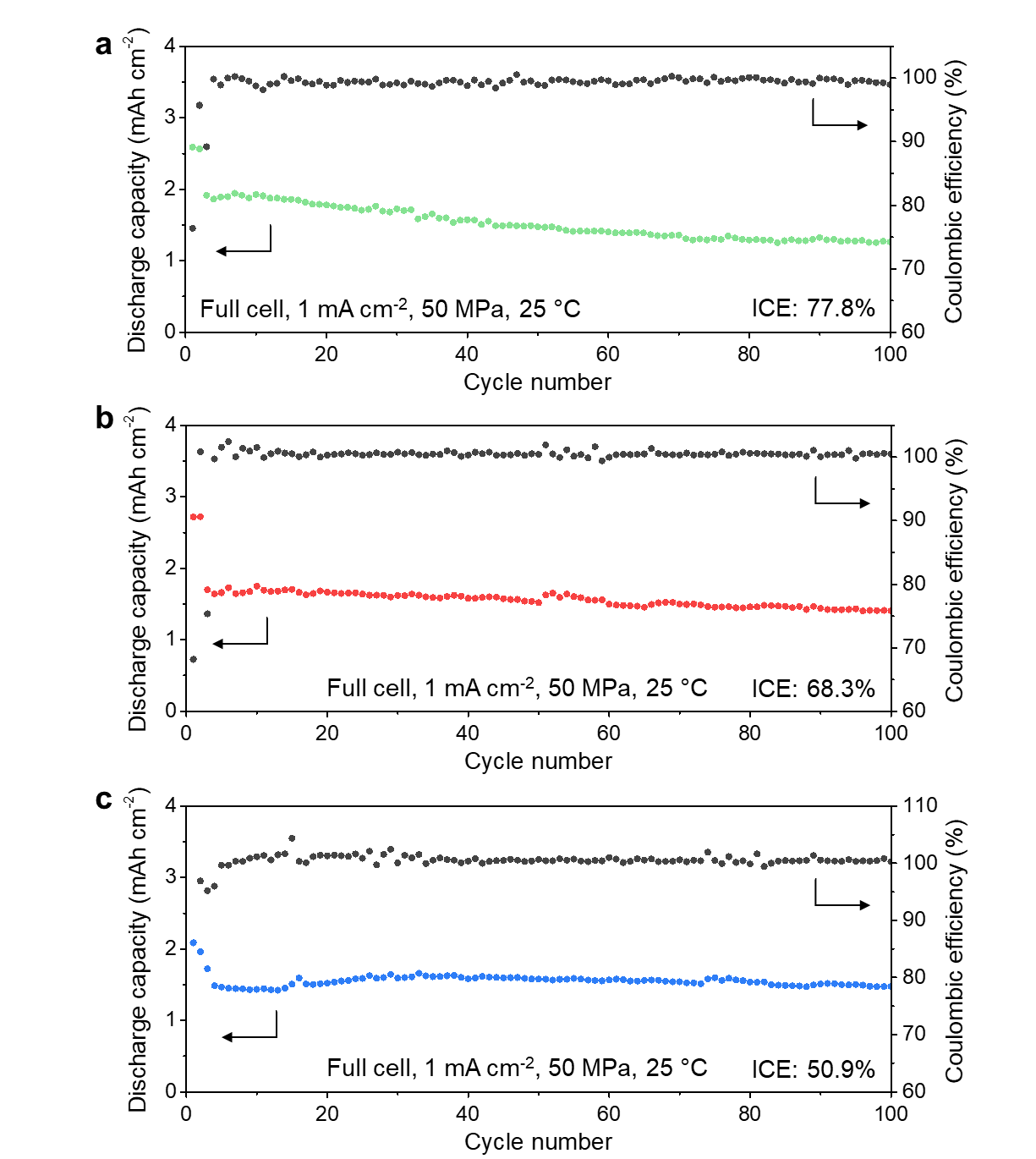


**Figure S12. Galvanostatic cycling of pristine (non-prelithiated) Si, Al, and Sn electrodes in full cells with LiNi_0.6_Co_0.2_Mn_0.2_O_2_ cathodes and Li_6_PS_5_Cl SSE.** **a**, Si. **b**, Al. **c**, Sn. The current density was 0.2 mA cm^-2^ for the first two cycles and 1.0 mA cm^-2^ for subsequent cycles. The cathode loading was 4.0 mAh cm^-2^. The stack pressure was 50 MPa.


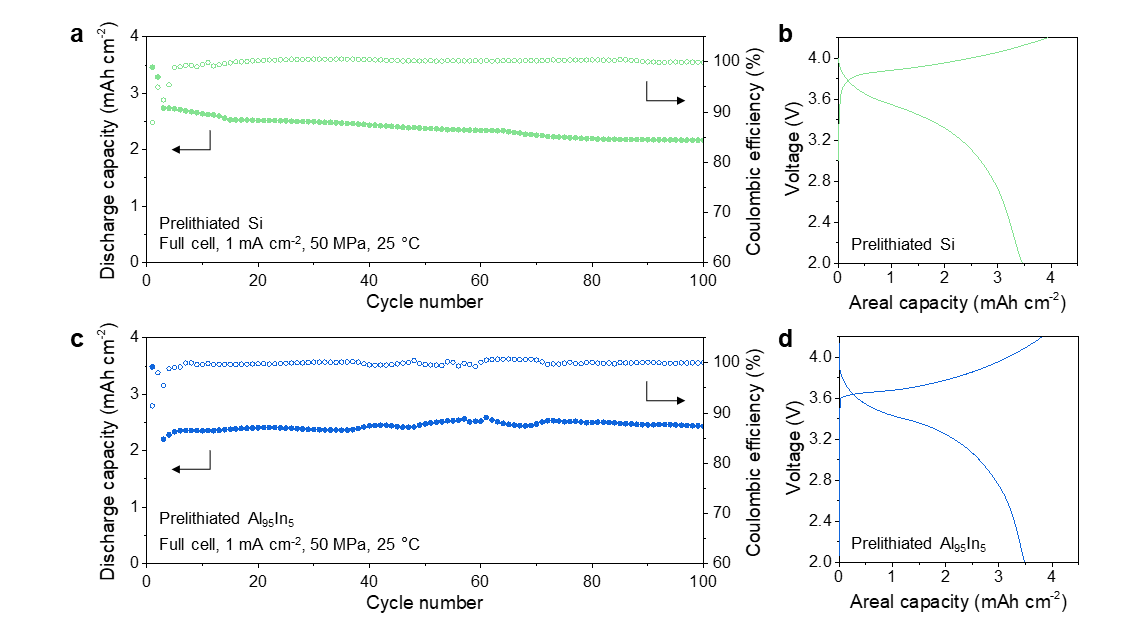


**Figure S13. Electrochemical performance of prelithiated electrodes in full cells with LiCoO_2_ (LCO) cathodes.** **a**, Areal capacity with cycle number during galvanostatic cycling of a cell containing a prelithiated Si anode and a LiCoO_2_ cathode. **b**, First-cycle voltage curve from the cycling test in (**a**). **c**, Areal capacity with cycle number during galvanostatic cycling of a cell containing a prelithiated Al_95_In_5_ foil anode and a LiCoO_2_ cathode. **d**, First-cycle voltage curve from the cycling test in (**c**). The current density was 0.2 mA cm^-2^ for the first two cycles and 1.0 mA cm^-2^ for subsequent cycles. The cathode loading was 4.0 mAh cm^-2^. The stack pressure was 50 MPa.


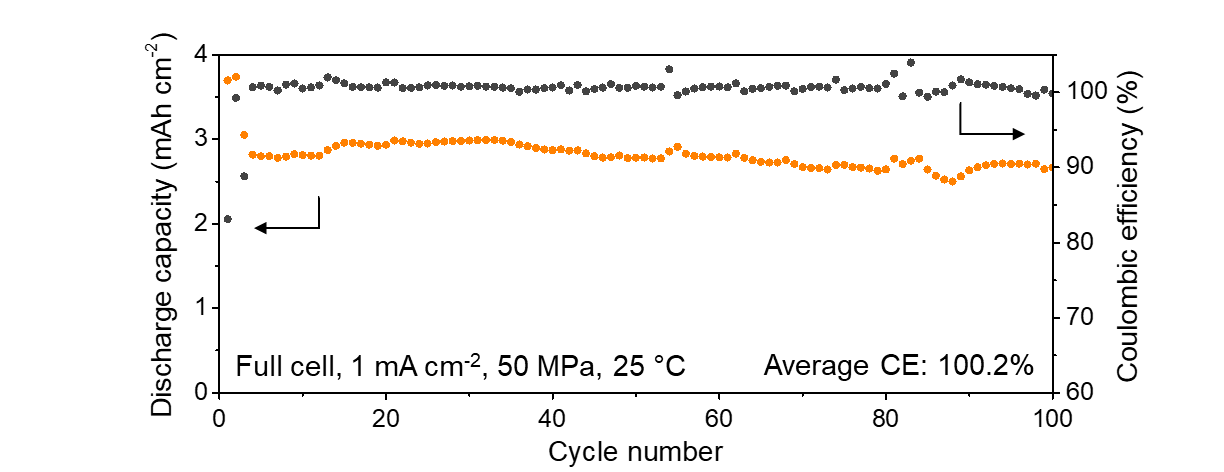


**Figure S14. Galvanostatic cycling of the prelithiated Al_95_Bi_5_ electrode in a full cell with LiNi_0.6_Co_0.2_Mn_0.2_O_2_ cathode and Li_6_PS_5_Cl SSE.** The current density was 0.2 mA cm^-2^ for the first two cycles and 1.0 mA cm^-2^ for subsequent cycles. The cathode loading was 4.0 mAh cm^-2^. The stack pressure was 50 MPa.


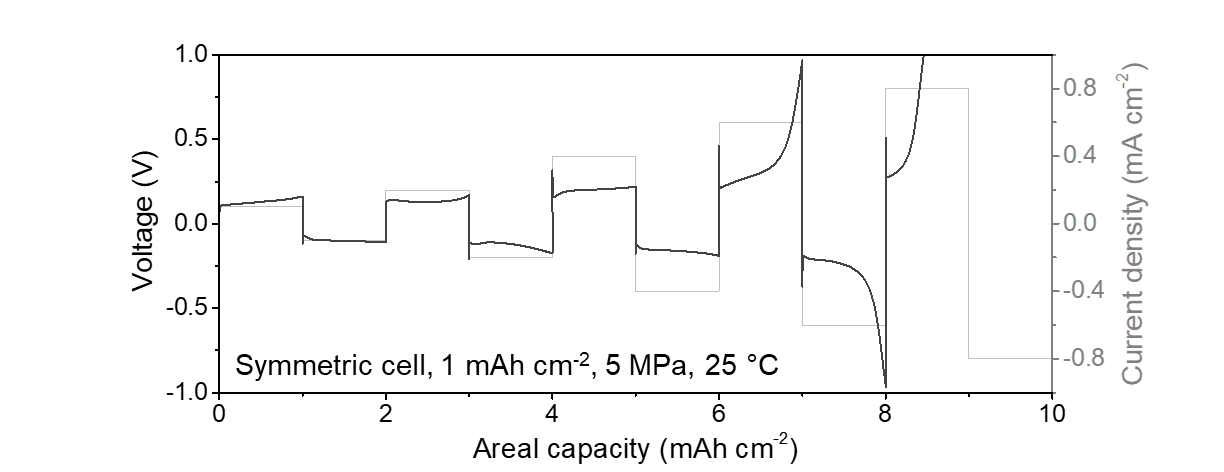


**Figure S15. CCD test of the prelithiated Al_95_In_5_ foil electrode in a symmetric cell at 5 MPa.**

**Table S2.** Electrochemical performance of alloy anodes in full-cell SSBs from literature.

| Anode | Prelithiation | Cathode loading  (mAh cm^-2^) | Current density (mA cm^-2^) | ICE (%) | Areal capacity  (mAh cm^-2^), number of cycles | Stack pressure  (MPa) | Ref. |
| --- | --- | --- | --- | --- | --- | --- | --- |
| Micro Si particle | No | 5 | 5 | 76 | 2.0  (500 cycles) | 50 | S1 |
| Nano Si particle | No | 2.4 | 0.24 | 50 | 1.15  (100 cycles) | 20 | S2 |
| Si particle | No | 2.2 | 0.7 | 82 | 1.2  (1000 cycles) | 50 | S3 |
| Lithiated  graphite/Si  particle | Yes | 3 | 1.5 | 84.8 | 1.7  (125 cycles) | 50 | S4 |
| Si particle | No | 4.3 | 0.43 | 82 | 2.2  (100 cycles) | 50 | S5 |
| Si particle | No | 1.28 | 0.15 | 85 | 1.5  (150 cycles) | 50 | S6 |
| Nano Si@LiAlO_2_ | No | 2 | 1 | 71 | 0.7  (150 cycles) | 60 | S7 |
| Prelithiated Si particle | Yes | 3 | 1.5 | 82 | 1.5  (300 cycles) | 120 | S8 |
| Nano Si particle | No | 3 | 0.15 | 76 | 2.7  (20 cycles) | 150 | S9 |
| Li_0.7_Si | Yes | 5 | 2.5 | 77.9 | 2.5  (2600 cycles) | 250 | S10 |
| Al_95_In_5_ foil | No | 5.8 | 0.8 | 85 | 2.0  (100 cycles) | 24 | S11 |
| Li-Al | Yes | 10 | 2 | - | 3.8  (50 cycles) | 70 | S12 |
| Li-Al | Yes | 2.88 | 0.72 | 93.1 | 2.0  (300 cycles) | 100 | S13 |
| Al foil | Yes | 4.0 | 1.0 | 84.2 | 2.3  (100 cycles) | 50 | This work |
| Micro Si particle | Yes | 4.0 | 1.0 | 85.8 | 2.3  (100 cycles) | 50 | This work |
| Sn foil | Yes | 4.0 | 1.0 | 84.5 | 2.0  (100 cycles) | 50 | This work |
| Al_95_In_5_ foil | Yes | 4.0 | 2.0 | 85.3 | 1.9  (500 cycles) | 50 | This work |
| Al_95_Bi_5_ foil | Yes | 4.0 | 1.0 | 83.1 | 2.7  (100 cycles) | 50 | This work |
| In-coated  Al_95_In_5_ foil | Yes | 4.0 | 1.0 | 84.3 | 1.5  (500 cycles) | 5 | This work |
| In-coated  Al_95_In_5_ foil | Yes | 4.0 | 0.5 | 85.0 | 1.7  (400 cycles) | 2 | This work |

**SUPPLEMENTARY REFERENCES**

1. Tan, D. H. S. *et al.* Carbon-free high-loading silicon anodes enabled by sulfide solid electrolytes. *Science* **373**, 1494–1499 (2021).

2. Han, S. Y. *et al.* Stress evolution during cycling of alloy-anode solid-state batteries. *Joule* **5**, 2450–2465 (2021).

3. Cao, D. *et al.* Long‐cycling sulfide‐based all‐solid‐state batteries enabled by electrochemo‐mechanically stable electrodes. *Adv. Mater.* **34**, 2200401 (2022).

4. Lee, J. *et al.* Dry pre‐lithiation for graphite‐silicon diffusion‐dependent electrode for all‐solid‐state battery. *Adv. Energy Mater.* **13**, 2300172 (2023).

5. Huo, H. *et al.* Chemo-mechanical failure mechanisms of the silicon anode in solid-state batteries. *Nat. Mater.* **23**, 543–551 (2024).

6. Yamamoto, M., Terauchi, Y., Sakuda, A., Kato, A. & Takahashi, M. Effects of volume variations under different compressive pressures on the performance and microstructure of all-solid-state batteries. *J. Power Sources* **473**, 228595 (2020).

7. Xu, X. *et al.* Nano silicon anode without electrolyte adding for sulfide‐based all‐solid‐state lithium‐ion batteries. *Small* **19**, 2302934 (2023).

8. Fan, Z. *et al.* In-situ prelithiation of electrolyte-free silicon anode for sulfide all-solid-state batteries. *eTransportation* **18**, 100277 (2023).

9. Cao, D. *et al.* Unveiling the mechanical and electrochemical evolution of nanosilicon composite anodes in sulfide‐based all‐solid‐state batteries. *Adv. Energy Mater.* **13**, 2203969 (2023).

10. Zhou, L. *et al.* Li_3–_*_x_*Zr*_x_*(Ho/Lu)_1–_*_x_*Cl_6_ solid electrolytes enable ultrahigh-loading solid-state batteries with a prelithiated Si anode. *ACS Energy Lett.* **8**, 3102–3111 (2023).

11. Liu, Y. *et al.* Aluminum foil negative electrodes with multiphase microstructure for all-solid-state Li-ion batteries. *Nat. Commun.* **14**, 3975 (2023).

12. Huang, Y., Shao, B. & Han, F. Li alloy anodes for high-rate and high-areal-capacity solid-state batteries. *J. Mater. Chem. A* **10**, 12350–12358 (2022).

13. Fan, Z. *et al.* Long‐cycling all‐solid‐state batteries achieved by 2D interface between prelithiated aluminum foil anode and sulfide electrolyte. *Small* **18**, 2204037 (2022).
